# Supplementary material for: Chromothripsis during telomere crisis is independent of NHEJ, and consistent with a replicative origin
Source: Genome Res. 2019 May;29(5):737–49. doi: 10.1101/gr.240705.118 (PMC6499312; doi:10.1101/gr.240705.118)
Supplement: Supplemental Material [file supp_gr.240705.118_Supplemental_file_1.zip › contigs/annotated_contigs/DB57/contig.2.DB57_length_621_mean_cov_3.57487922705.docx]

**DB57_length_621_mean_cov_3.57487922705**

```
TTCTTGAGACAGAAACAGCAGAACTTGTGCTTGGCTCATCCCTTCTATCCCTTTGGCCAGTATTCCATGTATATTAAAATATTTTCATT
 >chr7:141710215-141710546 + E=2e-178
AACAATAATTTCCAGAAGAGGCATTTCATTTCCATTCTTGCAGCCATCCCTAGATCATCATTTCCTCATGCTTGGGTTGCTCCAGGTGC

CCCTTGGCATCCCTTCTGAATTCCATTTCTCCTATCCATTCCGTTACATGTGGGATTAGAATTTCTCTTTTTTACATCCAACTTTTGTT

ATTATTTCTACCCAACTTCAGTACATATAGTGGTTTATTACTGTACGTTGAGCCAAGTTCAA|AC|TTAGAGCAATTTCAGTTAGACTG
                                                               >chr7:141712052-14171234
GTGCTTTGGCTGGAATGTGCCCCCTCCAAAATTCACGTGTTGCCAGTATGTTAGTATTAACAGTGGGGCCTTTAAGAGGTGATTAGGCC
4 - E=5e-164
ATGAAGCCTCCTCCCAGACAAAGGGAATTAGGTGCCCTTATAAAGGAGACAGTGGGCTGTCTCTTGCTGTCTACGTGCTGCCATGTGAG

GATGCAGCAAGAAGGTCCTCACAAGATGCCAGCACCTTGGTCTTGTTCTTTCTAGTCTCCATCCATAACTATGATAAGTAAATTTCTGT
```
